# Inclusions in diamonds probe Earth’s chemistry through deep time

**DOI:** 10.1038/s42004-022-00627-1

**Published:** 2022-01-26

**Authors:** Matteo Alvaro, Ross John Angel, Fabrizio Nestola

**Affiliations:** 1 Dipartimento di Scienze della Terra e dell’Ambiente, Università di Pavia, Via A. Ferrata, 1, 27100 Pavia, Italy; 2 IGG CNR, Via Giovanni Gradenigo 6, 35131 Padova, Italy; 3 Dipartimento di Geoscienze, Università degli Studi di Padova, Via G. Gradenigo 6, I-35131 Padova, Italy

**Keywords:** Geochemistry, Phase transitions and critical phenomena

## Abstract

Most of our knowledge about the chemical composition of the Earth’s interior is primarily retrieved by indirect observations, experiments and calculations that are limited to simple compositions. Here, the authors present the investigation of inclusions trapped in super deep diamonds as an alternative source of a wealth of information on the chemical state of the Earth’s interior through time.

The exploration of the interior of our planet has traditionally been through studies of natural rock samples found on or near the surface, supplemented by experiments on analogue materials synthesised and tested in our laboratories under a variety of externally controlled conditions chosen to simulate those expected in the Earth’s interior. Modern analytical instruments now provide new opportunities to measure significantly smaller samples or portions of samples down to the sub-micrometre or nano-scale. We have therefore progressively moved the subject of our investigations from the scale of a rock (centimeters to tens of centimeters) to the scale of impurities (that we call “inclusions”) that are commonly found trapped inside individual mineral crystals^1^. As our ability to interrogate mineral inclusions at finer and finer scales has developed, they have yielded more and more information about the chemistry of the Earth’s deep interior and how that is connected to, and influences, the biosphere and the surface of our planet.

## Sampling the Earth’s interior

What we know about the chemical composition of the deep Earth is mostly retrieved by indirect geophysical observations (e.g., seismic wave velocities and how they change with depth), by high-pressure and high-temperature laboratory experiments which are limited to simple compositions of the starting materials and ab initio calculations that are also very limited in terms of the compositional variability that can be explored. Indeed, most of what we know about the composition of the deep Earth is subject to sometimes extreme extrapolation. On the other hand, super-deep diamonds can trap a large variety of “real and direct” samples from even 1000 km deep in our planet. Measurements of the chemical composition of these pristine inclusions thus provide a more realistic view on the chemical composition of Earth’s interior. After encapsulation in the host mineral phase, inclusions are isolated from further change. They are transported back to the Earth’s surface protected by their host from further interaction with other minerals and fluids. Thus, unlike bulk rocks which are metamorphosed and modified after initial formation, inclusions can preserve the original chemical and isotopic signature of that part of the inner Earth in which they were originally formed and trapped.

**Fig. 1:**
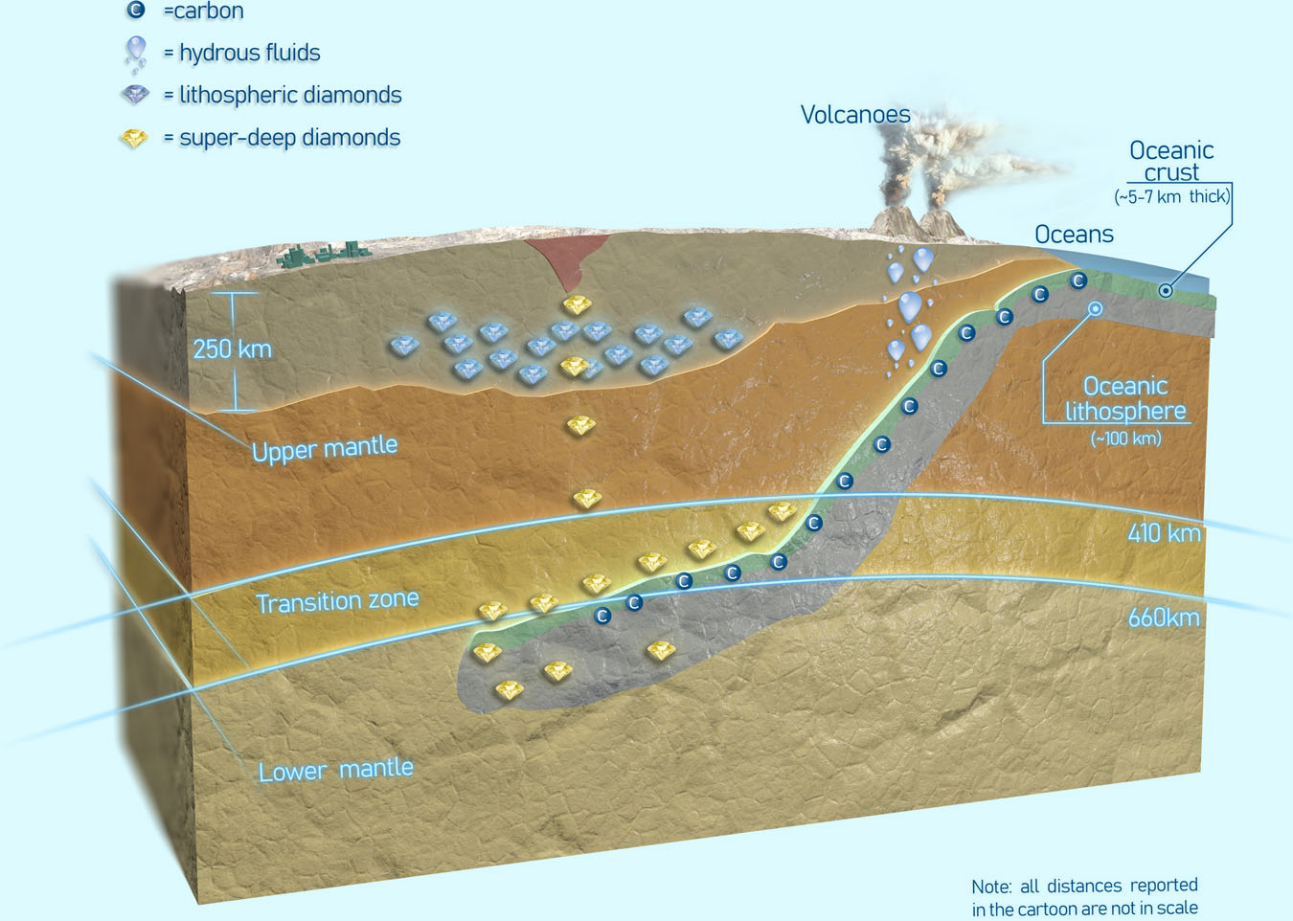
Diamonds journey through the Earth’s interior. Cartoon showing the growth zones for lithospheric diamonds (the first 250 km of depth) and super-deep diamonds (410–700 km depth). The grey part indicates a subducting slab that can penetrate into the lower mantle at depths greater than 660 km (cartoon by F. Chirico).

**Fig. 2:**
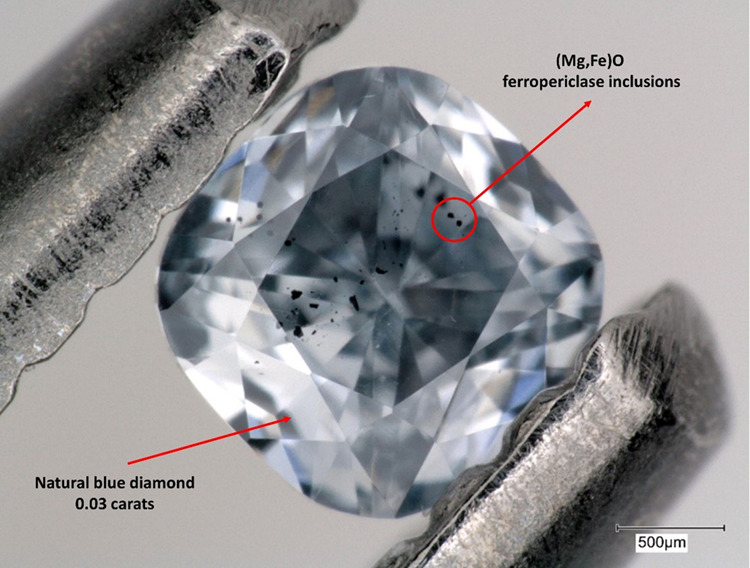
Natural blue diamond. Natural blue diamond containing black inclusions of ferropericlase (Mg,Fe)O indicating a minimum depth of formation not less than 15–16 GPa (about 450–480 km depth). The blue colour, as in the Hope diamond from the Smithsonian Institution, is due to the presence of very small amount of boron, B, which substitutes for carbon within the diamond structure. The presence of boron within a super-deep diamond demonstrates how our planet is efficient at recycling light elements crucial for life from the surface to the deepest regions of the Earth (Photo by Evan M. Smith/GIA).

Because of its wide stability in pressure and temperature, and its peculiar strength and chemical resistance, diamond is the most important carrier of inclusions from great depths in the Earth to the surface^2–5^. Inclusions can be trapped within diamonds from the shallow lithosphere at between 120 and 220 km in depth (lithospheric diamonds) as well as at far greater depths of 700–800 km in “super deep diamonds” (Fig. 1). From the investigation of these inclusions, we can therefore gain a wealth of information on the chemical composition, redox state, and stable phase assemblages of the Earth’s interior and its geodynamic processes from the shallowest regions to great depths (Fig. 1). For example, when we study super-deep diamonds with a clear surface carbon signature^2^ and the rare blue colour due to the presence of boron (B) impurities within the diamond structure^4^ then we are holding in our hands diamonds (Fig. 2) formed by light elements that in several millions of years travelled from the Earth’s surface down to the lower mantle and back to our hands to be studied in our laboratories. This continuous exchange between the inner portion of our planet and the biosphere is a substantial portion of the cycle of these light elements refuelling life at the surface of the Earth. Apart from the isotopes of C and its few trace chemical substituents such as B, the diamonds themselves carry little or no chemical information about the depth or environment in which they grew. That is provided by the chemical composition of the inclusions within the gemstones. Traditionally, that has been obtained through extraction of the inclusions. But extraction destroys much other information; fifty years of inclusion extraction never detected the original deep-earth fluids recently shown by in-situ spectroscopy to be present in many inclusions^6^. Extraction of inclusions also destroys a host of other information that can be obtained by exploiting their mechanical behaviour while still trapped in their host. To use the chemical information from inclusions we need to know the depths in the Earth at which the diamonds grew, and when they grew.

## The problem of depth

The contrast in elastic properties between the inclusion and the host diamonds can be exploited to calculate the depth of formation of the diamond^7,8^. The basic idea is that inclusions trapped at depth in the Earth are not free to expand but are constrained by the stiffer diamond during ascent to the Earth’s surface. Thus, even when the diamond is at room pressure, the trapped and constrained inclusion remains at a higher pressure. This residual stress can be measured in the lab if the inclusion is still trapped and preserved in its host. If the system did not undergo any non-elastic change, then the residual stress (or better strain) state of these extremely precious materials can be directly related to the depth at which they have been trapped into their hosts^7,9^. This is known as elastic barometry. It is based on an old idea^10^ that only recently has been improved and extended with modern tools and developments in the precision by which we can measure strains in microscopic inclusions by Raman spectroscopy^11^ and Xray diffraction^12^.

It is relatively straightforward to determine the pressure during the growth of shallow “lithospheric diamonds” from the stress state of their inclusions^13^, in the same way depths can be determined from inclusions in rocks from the shallower crust or subduction zones^1^. But when geologists want to explore the deeper Earth where super deep diamonds are formed, elastic geobarometry alone is no longer sufficient to provide the answers, because the much higher T and P at those depths leads to significant plastic deformation of the diamonds that modifies the stress state of the inclusions during their voyage to the Earth’s surface. The challenge is that our knowledge of the plasticity of diamond and how quickly it can flow to reset the inclusion stresses is extremely limited. The best that can be done is to make conservative estimates of the rates of this process and thus obtain a minimum P, T and depth at which the inclusion was trapped. Measurements of ferropericlase (Mg,Fe)O inclusions (Fig. 2) then yield the intriguing result that their minimum pressure of formation is 15.7 GPa at 1830 K (i.e. about 450 ± 70 km depth assuming lithostatic load), and probably represents the depth at which diamond deformation ceased during ascent within the Earth. If so, such diamonds may actually carry samples from the lower mantle of the Earth and provide information on element recycling from the surface to the more than 700km deep.

## The problem of age

Natural diamonds carry no chemical or isotopic signature that can currently be used to date the growth of the diamond. Thus the other crucial information that enables understanding rock history and thus cycling of elements in the Earth’s interior is isotopic dating of the inclusions in diamond, again made possible by significant advances in the capabilities to measure the isotropic and chemical composition of such small samples^14^. While shallower lithospheric diamonds have been very often dated thanks to their mineral inclusions, super-deep diamonds have hardly been dated at all. One of the first studies^15^ that reported a direct age determination of a super-deep diamond has been followed by more recent work^16^ that confirmed that super-deep diamonds appear to be much younger than shallower diamonds. Indeed, the only super-deep diamond directly dated provided an age of about 100 Ma compared to ages of lithospheric diamonds that range between 1 and 3 Ga^14^. Is this contrast real? Or is it just an artefact due to the absence of ages for super-deep diamonds? If super-deep diamonds do turn out to be as old as lithospheric diamonds, then such extremely rare samples could really represent our only window on the deepest regions of planet Earth from the beginning of its history. This has profound implications for the understanding of the development of the large-scale convection and structures (crust, upper mantle, transition zone and lower mantle, Fig. 1) of the Earth, and the element exchange and recycling between them over time.

## Outlook

Geoscience is still faced by many significant unknowns concerning the chemistry of the deep Earth; What is the detailed composition of the Earth’s core, and how did it evolve as a result of differentiation of the early Earth? How did the primaeval atmosphere evolve from out-gassing of the solid Earth? How homogeneous is the chemistry of the solid Earth, and what are the length scales of the inhomogeneities? Answers to these questions require direct information about the chemical composition of the deep Earth over time, and that can probably only be obtained from the inclusions trapped within diamonds at the time of their growth. In combination with isotopic dating now becoming possible on such rare and small samples, the determination of the stress states of inclusions and the deformation properties of diamond has the potential to yield fundamental data (dates and places) on the history and evolution of the detailed chemistry and element recycling in planet Earth and its impact on the biosphere. Finally, we will be able to explore the evolution of chemistry in deep time.
